# Suppression of bile acid synthesis as a tipping point in the disease course of primary sclerosing cholangitis

**DOI:** 10.1016/j.jhepr.2022.100561

**Published:** 2022-08-18

**Authors:** Peder Rustøen Braadland, Kai Markus Schneider, Annika Bergquist, Antonio Molinaro, Anita Lövgren-Sandblom, Marcus Henricsson, Tom Hemming Karlsen, Mette Vesterhus, Christian Trautwein, Johannes Roksund Hov, Hanns-Ulrich Marschall

**Affiliations:** 1 Norwegian PSC Research Center, Department of Transplantation Medicine, Oslo University Hospital, Oslo, Norway; 2 Institute of Clinical Medicine, University of Oslo, Oslo, Norway; 3 Research Institute of Internal Medicine, Division of Surgery, Inflammatory Diseases and Transplantation, Oslo University Hospital, Oslo, Norway; 4 Department of Medicine III, University Hospital RWTH Aachen, Aachen, Germany#; 5 Department of Microbiology, Perelman School of Medicine, University of Pennsylvania, Philadelphia, PA, USA; 6 Unit of Gastroenterology and Rheumatology, Department of Medicine Huddinge, Karolinska Institutet, Karolinska University Hospital, Stockholm, Sweden#; 7 Department of Molecular and Clinical Medicine/Wallenberg Laboratory, Sahlgrenska Academy, University of Gothenburg, Gothenburg, Sweden#; 8 Department of Medicine, Section of Gastroenterology and Hepatology, Sahlgrenska University Hospital Gothenburg, Gothenburg, Sweden; 9 Department of Laboratory Medicine, Karolinska Institutet, Stockholm, Sweden; 10 Section of Gastroenterology, Department of Transplantation Medicine, Oslo University Hospital, Oslo, Norway; 11 Department of Medicine, Haraldsplass Deaconess Hospital and Department of Clinical Science, University of Bergen, Bergen, Norway

**Keywords:** Cholestasis, Ursodeoxycholic acid, Liver transplantation, Liver transplantation-free survival, Cholestatic liver disease, 7α-Hydroxy-4-cholesten-3-one, C4, Biliary disease, liver, AOM, Amsterdam–Oxford model, ASBT, apical sodium-dependent bile acid cotransporter, C4, 7α-hydroxy-4-cholesten-3-one, c-index, concordance index, CYP7A1, cytochrome P450 family 7 subfamily A member 1, FGF19, fibroblast growth factor 19, FXR, farnesoid X receptor, GUDCA, glycooursodeoxycholic acid, HR, hazard ratio, IBAT, ileal bile acid transporter, MELD, model for end-stage liver disease, PBC, primary biliary cholangitis, PSC, primary sclerosing cholangitis, STROBE, Strengthening the Reporting of Observational Studies in Epidemiology, TUDCA, tauroursodeoxycholic acid, UDCA, ursodeoxycholic acid, UPLC-MS/MS, ultraperformance liquid chromatography–tandem mass spectrometry

## Abstract

**Background & Aims:**

Farnesoid X receptor (FXR) agonists and fibroblast growth factor 19 (FGF19) analogues suppress bile acid synthesis and are being investigated for their potential therapeutic efficacy in cholestatic liver diseases. We investigated whether bile acid synthesis associated with outcomes in 2 independent populations of people with primary sclerosing cholangitis (PSC) not receiving such therapy.

**Methods:**

Concentrations of individual bile acids and 7α-hydroxy-4-cholesten-3-one (C4) were measured in blood samples from 330 patients with PSC attending tertiary care hospitals in the discovery and validation cohorts and from 100 healthy donors. We used a predefined multivariable Cox proportional hazards model to evaluate the prognostic value of C4 to predict liver transplantation-free survival and evaluated its performance in the validation cohort.

**Results:**

The bile acid synthesis marker C4 was negatively associated with total bile acids. Patients with fully suppressed bile acid synthesis had strongly elevated total bile acids and short liver transplantation-free survival. In multivariable models, a 50% reduction in C4 corresponded to increased hazards for liver transplantation or death in both the discovery (adjusted hazard ratio [HR] = 1.24, 95% CI 1.06–1.43) and validation (adjusted HR = 1.23, 95% CI 1.03–1.47) cohorts. Adding C4 to established risk scores added value to predict future events, and predicted survival probabilities were well calibrated externally. There was no discernible impact of ursodeoxycholic acid treatment on bile acid synthesis.

**Conclusions:**

Bile acid accumulation-associated suppression of bile acid synthesis was apparent in patients with advanced PSC and associated with reduced transplantation-free survival. In a subset of the patients, bile acid synthesis was likely suppressed beyond a tipping point at which any further pharmacological suppression may be futile. Implications for patient stratification and inclusion criteria for clinical trials in PSC warrant further investigation.

**Lay summary:**

We show, by measuring the level of the metabolite C4 in the blood from patients with primary sclerosing cholangitis (PSC), that low production of bile acids in the liver predicts a more rapid progression to severe disease. Many people with PSC appear to have fully suppressed bile acid production, and both established and new drugs that aim to reduce bile acid production may therefore be futile for them. We propose C4 as a test to find those likely to respond to these treatments.

## Highlights

• The bile acid synthesis marker C4 associated negatively with bile acid levels in patients with PSC.
• Suppression of bile acid synthesis was likely nearly complete in advanced PSC.
• UDCA treatment contributed significantly to total circulating bile acids but did not appear to affect bile acid synthesis.
• Attempts to inhibit bile acid synthesis in patients with low C4 may be futile, and such drugs may be contraindicated.
• Patients with PSC and low circulating C4 had shorter liver transplantation-free survival in two independent cohorts.

## Graphical abstract

**Figure:**
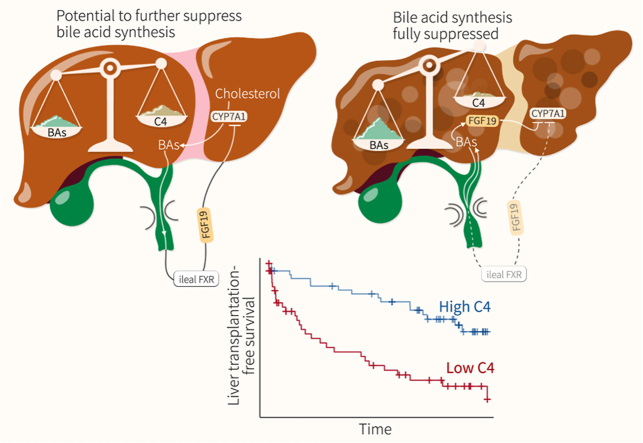

## Introduction

A hallmark of primary sclerosing cholangitis (PSC) is cholestasis caused by the formation of intrahepatic and/or extrahepatic bile duct strictures. No effective medical treatments are available, and death or liver transplantation occurs after a median of 13–21 years.^1^ Some patients live for years without symptoms, whereas others develop cancer or experience rapidly progressing liver disease early after diagnosis. This heterogeneous disease course is a major challenge in the clinical management of patients with PSC, and there is a lack of tools to evaluate prognosis or risk of complications or to predict response to therapy. Today’s best available tools indirectly reflect the fibrosis process, measuring liver stiffness with elastography or circulating fibrosis-related markers,[2], [3], [4] but markers of inflammation also predict disease activity in PSC.^5^

The clinical value of biomarkers of bile acid homeostasis is unexplored, despite studies showing hepatic and systemic accumulation of bile acids in cholestatic liver disease.^6^^,^^7^ In fact, the first large study showing an association between bile acid profiles and hepatic decompensation was only recently published.^8^ Biochemical footprints of cholestasis^8^ and degree of biliary changes^9^ are potent prognostic factors in PSC. Despite controversy about its efficacy, the bile acid ursodeoxycholic acid (UDCA) is the most commonly used drug, whereas drugs targeting the farnesoid X receptor (FXR)–fibroblast growth factor 19 (FGF19) axis, which regulates bile acid homeostasis, are actively pursued.^10^^,^^11^

Bile acids are synthesised in the liver and enter the intestine, where microbial modifications generate deconjugated and secondary bile acids. The majority of bile acids are reabsorbed in the terminal ileum and returned to the liver. In both the intestine and the liver, activation of the nuclear receptor FXR by bile acids leads to negative feedback and reduced transcription of the rate-limiting enzyme of bile acid synthesis cytochrome P450 family 7 subfamily A member 1 (CYP7A1). From the intestine, this is mediated via the release of the gut hormone FGF19. The FXR–FGF19 pathway is, therefore, an attractive target to influence bile acid synthesis. The activity of CYP7A1 can non-invasively be measured by the concentration of the circulating bile acid precursor 7α-hydroxy-cholesten-3-one (C4).^12^ Hence, C4 is a useful biomarker of the contribution of *de novo* synthesis to bile acid homeostasis in, for example, cholestatic liver diseases.

In a recent study investigating bile acid homeostasis in a murine PSC model and UDCA-naïve patients with PSC, we observed a negative association between levels of C4 and risk of liver transplantation or death.^13^ When using activators of the FXR–FGF19 axis, the bile acid synthesis and hence the C4 concentration will be reduced.^11^ Similarly, hepatic bile acid synthesis can intrinsically be suppressed in patients owing to cirrhosis. These patients with advanced disease are usually not included in clinical trials, and there are reports of severe adverse events in patients with cirrhosis, suggesting that therapeutic bile acid synthesis suppression is not beneficial or even harmful in these patients.^14^ In PSC, advanced disease is difficult to define, providing a strong rationale for investigating further the association seen between C4 levels and robust outcome measures observed in UDCA-naïve patients.^13^ We, therefore, investigated C4 in 2 different cohorts and 2 different laboratories, aiming to define the role of C4 as a prognostic factor and, in particular, the potential clinical usefulness of agents activating the FXR–FGF19 axis.

## Patients and methods

### Study design, participants, and samples

We used the cross-sectional sampling strategy. The Norwegian discovery cohort consisted of patients with PSC prospectively recruited at admission to the tertiary care hospital Oslo University Hospital, Rikshospitalet (Oslo, Norway) between 2008 and 2015. Samples from patients with PSC in the external validation cohort were collected at Karolinska University Hospital (Stockholm, Sweden) between 2008 and 2012. Healthy controls of age within the normal range of a general population with PSC were recruited from the Norwegian Bone Marrow Donor Registry (n = 100) (please refer to the Supplementary CTAT methods table).

PSC was diagnosed by accepted criteria, and hence, biological sample availability determined the sample sizes. We collected clinical follow-up data up until 2019.

We collected informed, written consent from all participating patients and healthy donors. The study was approved by the Regional Committee for Medical and Health Research Ethics in South-Eastern Norway (2011/2572 and 2015/2140) and the Regional Ethics Committee in Stockholm (2018/1111-32).

Serum and plasma samples were collected and kept according to a standardised procedure at each centre. The Norwegian plasma samples had not been thawed until the day of C4 analysis, whereas the Swedish serum samples had been frozen and thawed at least twice. Routine blood biochemistry results at baseline were collected from journal records and were temporally matched with blood samples used for bile acid profiling and C4 measurement for all but 2 patients (0.5%) where samples were taken 1 month apart. All relevant clinical and demographic data (Table 1) were collected from patient journals.

**Table 1:** **Baseline, non-imputed clinical, and biochemical characteristics of healthy controls and patients included in the study**.

Variable	Healthy controls	PSC	
	Norway, n = 100	Discovery, Norway, n = 191	Validation, Sweden, n = 139
Sex, female	41 (41%)	40 (21%)	44 (32%)
Age at sampling [min–max]	40 [28–56]	41 [16–72]	42 [21–77]
Inflammatory bowel disease, any	—	139 (74%)	103 (75%)
Ulcerative colitis	—	96 (51%)	86 (62%)
Crohn’s disease	—	31 (16%)	16 (12%)
Indeterminate colitis	—	12 (6.3%)	1 (<1%)
Missing	—	2 (1%)	0 (0%)
Ursodeoxycholic acid treatment	—	71 (37%)	102 (77%)
Missing	—	0 (0%)	7 (5%)
Hepatobiliary cancer, any∗	—	8 (5%)	1 (<1%)
Cholangiocarcinoma	—	6 (3%)	1 (<1 %)
Gallbladder cancer	—	1 (<1%)	0 (0%)
Hepatocellular carcinoma	—	1 (<1%)	0 (0%)
Variceal bleeding	—	5 (2.6%)	2 (1.4%)
Ascites	—	20 (10%)	0 (0%)
Encephalopathy	—	2 (1%)	0 (0%)
Mayo PSC score [IQR]	—	0.26 [-0.45 to 1.39]	0.04 [-0.58 to 0.78]
Missing	—	2	4
AOM PSC score [IQR]	—	1.81 [1.3–2.51]	1.78 [1.44–2.31]
Missing	—	15 (8%)	7 (5%)

AOM, Amsterdam–Oxford model; PSC, primary sclerosing cholangitis.

∗ An additional 20 and 4 patients were diagnosed with cholangiocarcinoma, and 4 and 0 with gall bladder cancer, in the discovery and validation cohorts, respectively, during follow-up.

Amsterdam–Oxford PSC and Mayo PSC scores were calculated according to de Vries *et al.*^9^ and Kim *et al.*,^15^ respectively, and were modelled as continuous variables.

The study reporting adhered to the STROBE statement.

### Bile acid and C4 analyses

Bile acids and C4 were analysed using ultraperformance liquid chromatography–tandem mass spectrometry (UPLC-MS/MS). Plasma samples from the Norwegian cohort were run at the Wallenberglab Laboratory (Sahlgrenska University Hospital, Gothenburg, Sweden), and serum samples from the Swedish cohort were run at the Department of Clinical Chemistry at Karolinska University Hospital, Stockholm, Sweden (Supplementary material and CTAT methods table). Total UDCA and UDCA metabolite enrichment was calculated as (TUDCA [tauroursodeoxycholic acid] + GUDCA [glycooursodeoxycholic acid] + UDCA + isoUDCA [ursodeoxycholic acids]):total bile acids (Table S1).

### Handling of missing data

The nonimputed circulating UDCA concentrations could classify actual UDCA use with a sensitivity of 0.85 and specificity of 1.00 using a cut-off of 225 nmol/L, which we used to classify UDCA use for the 7 patients in the validation cohort that lacked these clinical records.

Missing values in UDCA, isoUDCA, GUDCA, and TUDCA were considered below the lowest detection limit as their concentrations are primarily determined by UDCA treatment and were hence imputed to the lowest detected value. For the remaining bile acids (Table S1), 7 and 10% of the samples had a missing value, but no single bile acid was missing in >37% of the samples. No sample had a missing C4 value. Of routine biochemical parameters used to compute composite risk scores, 7 and 1% were missing. Missing clinical and blood biochemical values in each cohort were imputed using K-nearest neighbours in the *DMwR* package (Data Mining with R, learning with case studies; Luis Torgo, CRC Press 2010). We used a weighted average based on the Euclidean distance to the case and a number-of-neighbours equal to the square root of the number of variables used. UDCA medication, sex, bile acids, routine blood biochemistry, and the outcome were used as predictors.

The total bile acid concentration was calculated as the sum of all bile acids (after exclusion and imputation as detailed above).

### Outcome definition

Liver transplantation or all-cause mortality was used as a composite endpoint for the time-to-event analyses. Of note, the median waiting times after listing for liver transplantation in the Nordic have generally been around 1–2 months.^16^^,^^17^ Patients who underwent a liver transplantation within 3 months (n = 22) were excluded because they likely had their blood drawn in conjunction with a referral for liver transplantation. Two patients in the validation cohort lacked event status and were excluded. In addition, 6 patients and 1 patient, from the discovery and validation cohorts, respectively, had hepatobiliary cancer diagnoses before sampling and were excluded. As a hepatobiliary cancer diagnosis can be a competing event to liver transplantation, we censored patients who were diagnosed during the study’s follow-up at their dates of a cancer diagnosis.

In sensitivity analyses, we tested the effect of (i) excluding those diagnosed with a hepatobiliary cancer diagnosis during follow-up and (ii) following them until they reached the outcome. All individuals with a hepatobiliary cancer diagnosis died within the follow-up without receiving a liver transplant.

### Statistical analyses

Adjusted *R*^2^ was calculated to test for linear regression model goodness of fit. Spearman’s correlation (*r*_s_) was calculated to evaluate bivariate trends of associations.

The functional forms for the exposure and covariates were inspected by fitting loess lines between their Martingale residuals and their linear predictions. Median follow-up was calculated using the reverse Kaplan–Meier method.

Discrimination was assessed using Harrell’s concordance index (c-index) and by inspection of Kaplan–Meier survival curves where patients were censored at 10-year follow-up. We were not aware of any *a priori* reported, biologically meaningful cut points for C4. Hence, for Kaplan–Meier plots, we categorised patients by C4 quartiles and assessed differences in survival using log-rank tests.

To evaluate the added value of C4 when nested with established risk scores, we prespecified our Cox proportional hazards model to include the composite PSC risk scores from the Mayo clinic^15^ or Amsterdam–Oxford^9^ as additive, continuous covariates. External validation of predictive accuracy (calibration) was assessed by visual inspection of predicted survival (event-free) probabilities plotted against observed event-free proportions using *rms::survest* and *rms::val.surv*^18^ and the *polspline::hare* function. The smoothed calibration curve reflects the correspondence between the predicted event-free probabilities (using the fitted time-to-event model) and the observed event-free fractions at specific time horizons. Goodness of fit of the survival models were evaluated using likelihood ratio χ^2^ tests. The internal validity of the survival models was evaluated using resampling validation with 200 bootstrap repetitions using the *rms::validate* function. All statistical analyses were done in R (R Foundation for Statistical Computing, Vienna, Austria).

## Results

### Systemic bile acid accumulation associates with suppression of bile acid biosynthesis in PSC

**Fig. 1:**
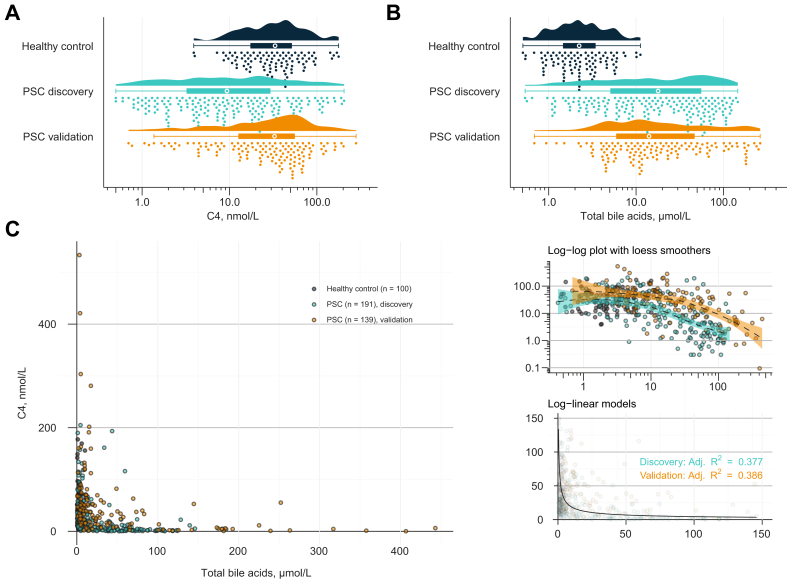
Cholestasis-driven suppression of bile acid synthesis is evident in PSC. (A and B) Circulating levels of C4 and total bile acids in healthy controls (n = 100) and patients with PSC in the discovery (n = 191) and validation (n = 139) cohorts. Individual data points, boxplots (white dots indicate the median), and probability densities are shown for each category. (C) Untransformed bivariate plot of C4 and total bile acids, coloured by whether the samples were drawn from individuals with or without PSC and cohort affiliation. Two outliers with C4 >300 nmol/L are not shown. To the upper right, the same data points are plotted on log_10_-transformed axes with smooth loess lines fitted for each cohort with PSC. To the lower right, an estimated log-linear regression model is plotted on the original scale (zoomed in for clarity) generated from all samples from patients with PSC pooled together. The adjusted R^2^ value from linear regressions fitted to each cohort separately is indented. C4, 7α-hydroxy-4-cholesten-3-one; PSC, primary sclerosing cholangitis.

The discovery cohort consisted of 191 patients with PSC and 100 healthy controls with similar age distributions (Table 1). The median (IQR) blood C4 concentration was 8.8 (2.75–26.8) nmol/L in the discovery cohort and 33.3 (17.3–51.8) nmol/L in the healthy controls (Wilcoxon rank-sum test *p* <0.0001) (Fig. 1A). Notably, the 5th, 50th, and 95th percentiles of C4 in the Norwegian healthy controls (9.1, 33.3, and 99.2 nmol/L, respectively) were similar to those previously found for Swedish healthy subjects (10.0, 33.9, and 102.3 nmol/L, respectively).^19^ In both healthy controls and patients with PSC, there was no evidence of a difference in C4 among individuals who were fasting and those who were postprandial at the time of blood draw (*p* = 0.11 for PSC and *p* = 0.81 for healthy controls).

To investigate the effect of systemic bile acids on bile acid synthesis, we compared total bile acid and C4 levels in the Norwegian samples (Table S1). As expected in cholestasis, patients with PSC had strongly elevated circulating bile acid levels, with a median (IQR) of 17.8 (4.8–55.6) μmol/L compared with a median (IQR) of 2.2 (1.4–3.4) μmol/L in healthy controls (Fig. 1B; Wilcoxon rank-sum test *p* <0.0001). Patients with PSC had a reduced deconjugated:conjugated bile acids ratio, particularly among UDCA-naïve patients (Table S2), which is congruent with biliary obstruction favouring hepatic accumulation rather than gut microbial enzymatic deconjugation. There was a clear deviance from linearity between C4 and total bile acids in blood sampled from patients with PSC (Fig. 1C). The best regression fit between C4 and total bile acids appeared to be a log-linear one (adjusted *R*^2^ = 0.38 and 0.39 in the discovery and validation cohorts, respectively). There was no evidence of a correlation between C4 and total bile acids in healthy controls (Spearman’s rank-order correlation *r*_s_ = 0.01), and in blood from patients with PSC, there was a tendency of a tapering in the increase in C4 at total bile acid concentrations within the normal range of healthy controls (Fig. 1C, top-right panel). In patients with PSC with higher bile acid concentrations, the absolute increase in suppression of bile acid synthesis tapered with increasing accumulation of systemic bile acids.

We next measured C4 levels in 139 serum samples collected from patients with PSC attending a tertiary care centre in Sweden (validation cohort; Table 1). These patients had overall less advanced liver disease with fewer complications such as variceal bleeding, ascites, and encephalopathy; lower median Mayo PSC scores; and a higher proportion of patients being treated with UDCA (102 [77%] *vs*. 71 [37%]). The median C4 level was 33.3 nmol/L (IQR 12.6–60.3 nmol/L), which was similar to the Norwegian healthy controls. However, there was a long tail of low C4 validation cohort samples. In fact, 31 (22%) of the samples had C4 levels below the fifth percentile of healthy controls. Furthermore, the same negative relationship between C4 and total bile acids was found in the validation cohort samples (Fig. 1C; *r*_s_ = −0.56, *p* <0.0001), which contrasted the mentioned lack of a trend of an association in the healthy controls. Hence, despite high median C4 levels, the validation cohort also represented a patient population with cholestasis with a bile acid-mediated, dose-dependent suppression of bile acid synthesis.

### UDCA has no discernible impact on bile acid synthesis

**Fig. 2:**
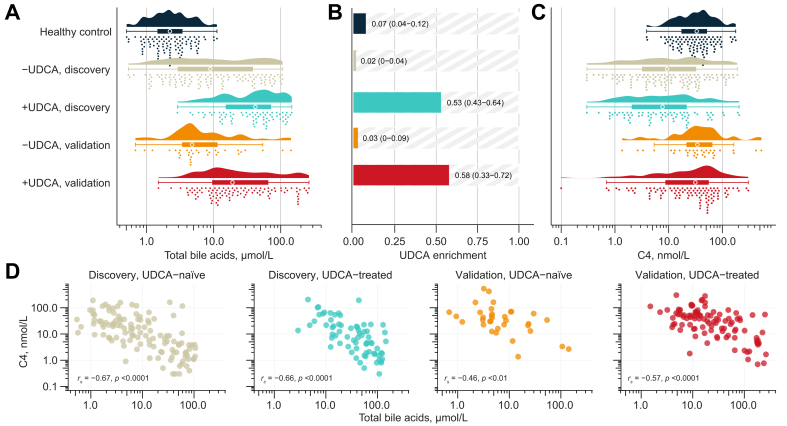
The limited apparent effect of UDCA on bile acid-mediated regulation of bile acid synthesis. (A) Concentrations of total bile acids in circulation, grouped by phenotype, cohort, and UDCA use. (B) Fractions of circulating UDCA and UDCA-derived bile acids relative to the total circulating bile acids (UDCA enrichment; UDCA + isoUDCA + GUDCA + TUDCA/total bile acids). Median enrichment (IQR) is annotated to the right of each bar. (C) Concentrations of C4 in circulation, grouped by phenotype, cohort, and UDCA use. (D) Bivariate log–log plots of C4 and total bile acids stratified by cohort affiliation and UDCA treatment status. The strengths of the associations between paired samples were tested using Spearman’s rank-order correlation (r_s_). C4, 7α-hydroxy-4-cholesten-3-one; GUDCA, glycooursodeoxycholic acid; TUDCA, tauroursodeoxycholic acid; UDCA, ursodeoxycholic acid.

Having bile acid profiles of both UDCA-naïve and UDCA-treated patients allowed us to explore the contribution of this drug to the total amount of circulating bile acids and its potential effect on FXR activation.^20^ There was a high concordance between UDCA use and circulating UDCA (Fig. S1 and Tables S1 and S2). Total bile acid levels were more than 4 times higher in the UDCA-treated than in the UDCA-naïve patients (Fig. 2A and Table S2), and expectedly, treated patients had a higher enrichment of UDCA and its metabolites (Fig. 2B). Although those constituted more than half of the circulating total bile acids in the patients taking UDCA, median bile acid synthesis was not different between those taking and those not taking UDCA (Fig. 2C). There were also no apparent differences in the associations between C4 and total bile acids by UDCA treatment status (Fig. 2D), and there were no interactions between C4 and UDCA enrichment in the prediction of total bile acid levels (linear regression, *p*_interaction_ >0.8).

### Patients with PSC with suppressed synthesis of bile acids experience shorter liver transplantation-free survival

Cholestasis-driven liver damage is believed to be a major cause of the complications of advanced PSC. We, therefore, evaluated whether circulating levels of C4 had an apparent association with liver transplantation-free survival and if it could improve prediction atop established risk prediction models in 2 independent populations with PSC, irrespective of UDCA use. Baseline characteristics of the patients eligible for survival analyses are shown in Table S3. The median follow-up was 9.17 and 9.47 years in the discovery and validation cohorts, respectively. Of 167 and 135 patients eligible for survival analyses, 62 (37%) and 40 (30%) had a recorded endpoint, respectively. The baseline survival in the discovery cohort was shorter than that in the validation cohort (Fig. S2; log-rank *p* <0.0001).

Fig. 2A shows the Kaplan–Meier curves for liver transplantation-free survival of patients in the discovery cohort categorised by C4 quartile boundaries. There was an apparent discrimination of the survival curves, where patients within the higher quartiles had longer survival (overall log-rank *p* <0.0001). To assess the predictive accuracy of C4 in an independent population, we used the same boundaries to categorise patients in the validation cohort (n = 136). Here, discrimination was also apparent, with a clearer separation of survival curves among patients with intermediate-level C4 (3.8–31.5 nmol/L), thus supporting the prognostic value of C4 in PSC.

To obtain a linear functional form, we modelled C4 as a multiplicative marker (log_2_). Increasing C4 associated with a lower crude hazard for reaching an endpoint in both cohorts (Table 2), corresponding to a 37% (95% CI 20–56%) and 47% (95% CI 27–67%) increased crude hazard when reducing the C4 concentration by 50% in the discovery and validation cohorts, respectively. C4 appeared linearly and negatively correlated with both Mayo PSC score (*r*_s_ = -0.69 and -0.55 in discovery and validation cohorts, respectively, both *p* <0.0001) and Amsterdam–Oxford PSC score (*r*_s_ = -0.60 and -0.40, both *p* <0.0001). Holding the Mayo PSC score constant, C4 remained associated with a reduced hazard for reaching an endpoint in both cohorts (Table 2). In the discovery cohort, a 50% reduction in C4 was then consistent with up to a 43% increase in the adjusted hazard for liver transplantation, with a hazard ratio (HR) point estimate corresponding to 1.23 (95% CI 1.06–1.43). The HRs and CIs were largely similar in the validation cohort (adjusted HR = 1.23, 95% CI 1.03–1.56), and replacing the Mayo PSC score with the Amsterdam–Oxford model (AOM) risk score yielded comparable results in both cohorts (Table 2).

**Table 2:** **Model estimates and performance metrics from univariate and multivariable Cox proportional hazards models for liver transplantation-free survival in the discovery and validation cohorts**.

Model	HR [95% CI]	*p* value	c-index	Δc-index∗	LR χ^2^†	LR χ^2^*p*
**Discovery cohort (n = 167, n events = 62)**						
C4	0.73 [0.64–0.83]	<0.0001	0.660		23.5	<0.0001
Mayo PSC score	1.67 [1.36–2.04]	<0.0001	0.694		22.7	<0.0001
AOM PSC score	2.47 [1.82–3.36]	<0.0001	0.706		33.6	<0.0001
C4 + Mayo PSC score	0.81 [0.70–0.94]	0.0052	0.694	0.000	8.10	0.0044
C4 + AOM PSC score	0.82 [0.72–0.93]	0.0016	0.726	0.020	8.02	0.0046

**Validation cohort (n = 135, n events = 40)**						
C4	0.68 [0.60–0.79]	<0.0001	0.706		25.2	<0.0001
Mayo PSC score	2.30 [1.74–3.02]	<0.0001	0.764		31.85	<0.0001
AOM PSC score	2.58 [1.75–3.79]	<0.0001	0.708		23.09	<0.0001
C4 + Mayo PSC score	0.81 [0.68–0.97]	0.0215	0.775	0.011	4.98	0.0256
C4 + AOM PSC score	0.76 [0.64–0.89]	0.0007	0.734	0.026	10.6	0.0011

AOM, Amsterdam–Oxford model; C4, 7α-hydroxy-cholesten-3-one; c-index, concordance index; HR, hazard ratio; LR, likelihood ratio; PSC, primary sclerosing cholangitis; UDCA, ursodeoxycholic acid. For multivariable models, adjusted HRs, 95% CIs, and *p* values are shown for the exposure (log_2_(C4)).

∗ The delta (Δ) c-index is the difference in c-index of the (full) model and the corresponding simple model.

† For multivariable models, the LR test compares goodness of fit of the nested (full) model to that of the simple model.

Adding C4 to a simple model consisting of Mayo PSC score alone improved the goodness of fit significantly (at an alpha level of 0.05), with likelihood ratio χ^2^ statistics of 8.10 (*p* = 0.004) and 4.98 (*p* = 0.026) in the two cohorts, suggesting that C4 may add value to predict future events. Adding C4 did not translate into any notable increments in the less sensitive c-index (Table 2). The added value of C4 was, however, more apparent when nested with the AOM score in both cohorts. Of note, there was no clear evidence of an interaction between C4 and UDCA treatment in neither the univariate nor nested (multivariable) Cox models in either cohort (*p*_interaction_ >0.15).

**Fig. 3:**
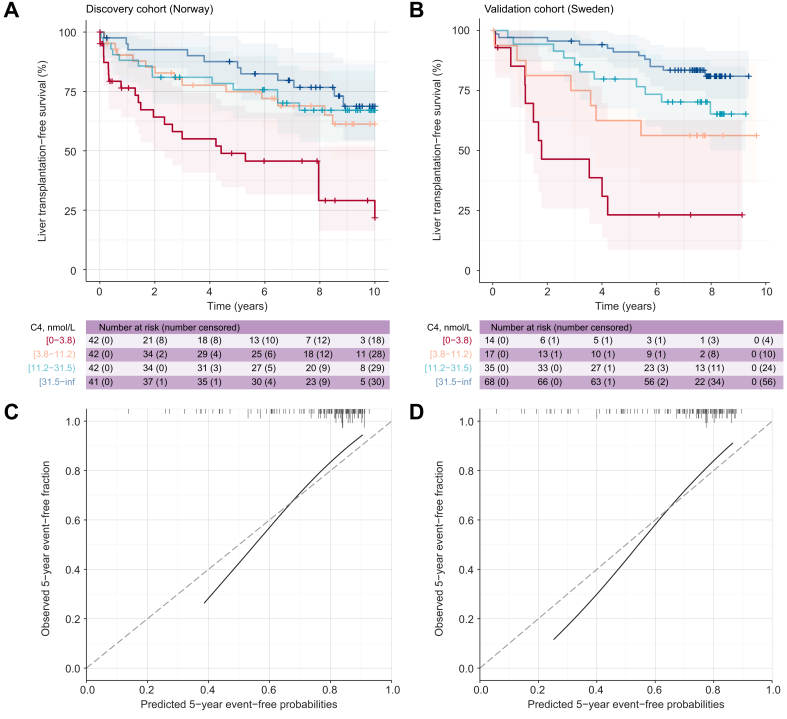
C4 associates with liver transplantation-free survival. Liver transplantation-free survival curves of patients with PSC in the (A) discovery cohort and (B) validation cohort, calculated using the Kaplan–Meier method. Patients were categorised by the boundaries determined by quartiles of C4 in the discovery cohort. In both cohorts, patients were censored at 10-year follow-up. Comparisons of the survival distributions were tested using log-rank tests (*p* values indented). The number at risk and number censored are shown for each indicated time point. Smoothed calibration curves illustrating the agreement between the estimated predicted event-free probabilities from the full Cox model (C4 + Mayo PSC score) and the observed event-free fractions in the external validation cohort at the (C) 5-year and (D) 8-year time horizons. The 45° dashed line indicates perfect calibration. One-dimensional histograms of the predicted event-free probabilities are shown on the top of each plot to illustrate their distributions. C4, 7α-hydroxy-4-cholesten-3-one; PSC, primary sclerosing cholangitis.

Upon resampling (internal) validation of our full model, there was a negligible decrease in the optimism-corrected c-index (0.687 *vs.* apparent c-index of 0.694) of the full model (C4 + Mayo PSC score), indicating that the model was not overly optimistic. The predicted survival probabilities of the full model calculated in the external validation cohort corresponded well with the observed survival rates (Fig. 3C and D). The smoothed calibration curves at the relevant 5- and 8-year time horizons indicated that the model, when applied to the external validation cohort, underestimated the fraction of patients experiencing the outcome where the estimated predicted risks were high. Calibration improved at higher predicted event-free probabilities, where most of the estimated probabilities were.

In sensitivity analyses, we found that ignoring hepatobiliary cancer diagnoses made during the follow-up and censoring these patients at their dates of death yielded estimates well comparable with the main analyses (discovery cohort, n = 167 with 79 events: adjusted HR for C4 = 0.83, 95% CI 0.73–0.95, *p* = 0.006; validation cohort, n = 135 with 44 events: adjusted HR for C4 = 0.82, 95% CI 0.69–0.97, *p* = 0.020). Excluding patients with hepatobiliary cancer diagnoses made during follow-up also gave similar estimates (discovery cohort, n = 150 with 62 events: adjusted HR for C4 = 0.81, 95% CI 0.70–0.94, *p* = 0.006; validation cohort, n = 131 with 40 events: adjusted HR for C4 = 0.81, 95% CI 0.68–0.97, *p* = 0.022). Finally, we specifically tested whether the association of C4 with survival was constant between UDCA users and nonusers by fitting interaction terms in the full Cox models. The interaction terms were not statistically significant at an alpha threshold of 0.05 (*p*_interaction_ = 0.86 in the Norwegian cohort and 0.19 in the Swedish cohort).

## Discussion

In the present study, we show in 2 large independent cohorts that as PSC becomes more advanced, bile acid synthesis is increasingly suppressed. From the apparent log-linear relationship between C4 and total bile acids, which appeared to be independent of UDCA use, it follows that the absolute increase in suppression of bile acid synthesis tapers with increasing accumulation of systemic bile acids. Hence, patients with PSC progressing beyond a tipping point where bile acid synthesis, in reality, is fully suppressed are unlikely to benefit from further pharmacological stimulation of the FXR–FGF19 axis. We find that low C4 associates with shortened liver transplantation-free survival also when modelled together with other, established prognostic factors in multivariable prediction models and show that these models generalise well in an independent patient cohort.

Apart from the preliminary data from a UDCA-naïve subset of the discovery cohort of the present study,^13^ the prognostic value of C4 in cholestatic liver diseases is largely unexplored. C4 was reduced in a study of bile acid homeostasis in 12 patients with PSC, out of whom 5 had intermediate to high Mayo risk scores and fully suppressed C4.^21^ In primary biliary cholangitis (PBC), another progressive cholestatic disease, the concentration of C4 was also reduced compared with that in healthy controls, particularly in patients with cirrhosis.^22^^,^^23^ In the present study, we show that C4 adds value to predict future events on top of established risk scores in PSC. Furthermore, the satisfactory calibration and discrimination in an external cohort indicates that our model is likely to both perform and generalise well in unseen patient cohorts.

Ongoing and previously performed drug trials with agents stimulating the FXR–FGF19–CYP7A1 axis have found drug-induced decreases in bile acids and C4.^11^ However, these studies did not include patients with PSC with advanced liver disease and intrinsically depressed bile acid synthesis.^11^^,^^24^^,^^25^ In such patients, further reduction of C4 following FXR–FGF19 activation is likely impossible,^21^ and the likelihood of a beneficial effect may be reduced, whereas the risk of adverse events may increase.^26^ Therefore, we propose to explore the use of C4 as a selection criterion to predict treatment response by drugs targeting FXR–FGF19 and also to monitor the effect of inhibitors of apical sodium-dependent bile acid cotransporters (ASBTs) expressed on ileal enterocytes (so-called ileal bile acid transporter [IBAT] inhibitors). Available C4 data of healthy Scandinavian populations,^13^^,^^19^ including this study, suggest that C4 values below their fifth percentile, that is, less than ∼5–9 nmol/L, reflect practically fully suppressed intrinsic bile acid synthesis and hence possibly a tipping point, whereby any additional means to suppress bile acid synthesis further will be futile. This should be further explored in carefully designed studies.

Of note, we found no evidence of an association between C4 and total bile acids among healthy controls, likely reflecting a steady state of bile acid synthesis. This appeared to also be the case among patients with PSC with total bile acids within the normal range (*i.e.* below 10 μmol/L), where the log-linear negative relationship tapered off. In line with a previous study,^27^ we did not see significant differences in C4 when comparing fasting and non-fasting individuals, which is useful for clinical implementation.

In our cohorts with PSC, patients treated with UDCA had elevated levels of circulating bile acids, mainly explained by increases in conjugated UDCA. There was, however, no difference in C4 concentrations in patients with PSC taking or not taking UDCA, providing no sign of a clinically relevant FXR-antagonistic effect, as has been suggested in obese individuals.^20^ Despite this, we cannot rule out the possibility that the C4 level in patients being administered UDCA was confounded by characteristics not possible to identify systematically by patient chart review (*e.g.* the indication for starting UDCA therapy).

Other limitations include the retrospective design, potential sampling bias at tertiary care centres, and the lack of data on fasting status for patients in the validation cohort. Although the predictive model generalised well to the external validation cohort, the model may not perform equally well on patients from other geographic regions or different care settings where additional confounders may need to be added to improve calibration. End-stage liver disease and disease complications with poor quality of life (*e.g.* recurrent cholangitis) were equally important as the main indications for PSC-related liver transplantation in Norway in 2013, although end-stage liver disease associated with higher model for end-stage liver disease (MELD) scores.^17^ Thus, our choice to use the composite endpoint of the earlier of either liver transplantation or death should be carefully interpreted. Still, the high degree of discrimination and calibration in 2 independent cohorts using distinct analytical instruments support the validity of the results. Finally, the lack of disease controls, which limits the generalisability of our observations to other cholestatic and non-cholestatic liver diseases, warrants similar studies also in such conditions.

### Conclusions

Taken together, we show that C4 may have clinical utility both as a sensitive marker of disease stage and to guide treatment strategies. Measuring blood C4 levels may, aside from monitoring treatment responses and serving as a potential counterindication for certain drugs, help identify patients with PSC who may need closer follow-up. As a response to cholestasis, many patients with PSC with advanced disease will have progressed beyond a ‘tipping point’, whereby bile acid synthesis is fully suppressed and any further therapeutic activation of the FXR–FGF19 axis will be futile. In contrast to complex bile acid profiles,^8^ C4 is easy to measure and interpret, and it is not impacted by the use of UDCA. Therefore, its clinical utility should be further explored in PSC and other cholestatic diseases.

## Financial support

PRB and JRH were funded by the 10.13039/100010663European Research Council (grant number 802544); HUM was funded by the 10.13039/501100004359Swedish Research Council (2019-01069) and the Swedish state under the agreement between the Swedish government and the county councils, and the ALF agreement (ALFGBG-ALFGBG-717231); KMS was supported by a 10.13039/501100001659Deutsche Forschungsgemeinschaft (DFG) research scholarship (SCHN 1626/1-1); and CT was supported by 10.13039/501100001659DFG (Project-ID 403224013 – CRC 1382). Aside from economic support, the funders had no role in the work that led to this manuscript.

## Authors’ contributions

Biochemical analyses: ALS, MH, AM. Data analyses and statistical analyses: PRB, KMS, HUM, JRH. Collection of patient material and clinical data: JRH, AB, MV, THK. Writing of the manuscript: PRB, KMS, JRH, HUM. Critical reading and editing of the manuscript and approval of the final version: PRB, KMS, AB, AM, ALS, MH, THK, MV, CT, JRH, HUM.

## Data availability statement

Adherence to national data protection laws prohibits us from unconditional sharing of individual participant data, but pseudonymised individual-level participant data of the variables reported in the present paper can be shared in agreement with the investigators, upon signing a material and data transfer agreement between the institutions and approval of necessary project amendments by the committees of research ethics.

## Conflicts of interest

JRH reports receiving consultant fees from Novartis and Orkla Health, lecture honoraria from Roche, and research funding from Biogen, none of which relate to this work. HUM reports receiving consultant fees from Calliditas, Mirum, and Zealand, and lecture honoraria from Albireo, none of which relate to this work. MV reports receiving lecture honoraria from Siemens Healthineers and Intercept, none of which relate to this work.

Please refer to the accompanying ICMJE disclosure forms for further details.

## References

[1] Boonstra K., Weersma R.K., van Erpecum K.J., Rauws E.A., Spanier B.W.M., Poen A.C. (2013). Population-based epidemiology, malignancy risk, and outcome of primary sclerosing cholangitis. Hepatology.

[2] Corpechot C., Gaouar F., El Naggar A., Kemgang A., Wendum D., Poupon R. (2014). Baseline values and changes in liver stiffness measured by transient elastography are associated with severity of fibrosis and outcomes of patients with primary sclerosing cholangitis. Gastroenterology.

[3] Vesterhus M., Hov J.R., Holm A., Schrumpf E., Nygård S., Godang K. (2015). Enhanced liver fibrosis score predicts transplant-free survival in primary sclerosing cholangitis. Hepatology.

[4] de Vries E.M.G., Färkkilä M., Milkiewicz P., Hov J.R., Eksteen B., Thorburn D. (2017). Enhanced liver fibrosis test predicts transplant-free survival in primary sclerosing cholangitis, a multi-centre study. Liver Int.

[5] Vesterhus M., Holm A., Hov J.R., Nygård S., Schrumpf E., Melum E. (2017). Novel serum and bile protein markers predict primary sclerosing cholangitis disease severity and prognosis. J Hepatol.

[6] Fischer S., Beuers U., Spengler U., Zwiebel F.M., Koebe H.G. (1996). Hepatic levels of bile acids in end-stage chronic cholestatic liver disease. Clin Chim Acta.

[7] Trottier J., Białek A., Caron P., Straka R.J., Heathcote J., Milkiewicz P. (2012). Metabolomic profiling of 17 bile acids in serum from patients with primary biliary cirrhosis and primary sclerosing cholangitis: a pilot study. Dig Liver Dis.

[8] Mousa O.Y., Juran B.D., McCauley B.M., Vesterhus M.N., Folseraas T., Turgeon C.T. (2021). Bile acid profiles in primary sclerosing cholangitis and their ability to predict hepatic decompensation. Hepatology.

[9] de Vries E.M., Wang J., Williamson K.D., Leeflang M.M., Boonstra K., Weersma R.K. (2018). A novel prognostic model for transplant-free survival in primary sclerosing cholangitis. Gut.

[10] Vesterhus M., Karlsen T.H. (2020). Emerging therapies in primary sclerosing cholangitis: pathophysiological basis and clinical opportunities. J Gastroenterol.

[11] Kowdley K.V., Vuppalanchi R., Levy C., Floreani A., Andreone P., LaRusso N.F. (2020). A randomized, placebo-controlled, phase II study of obeticholic acid for primary sclerosing cholangitis. J Hepatol.

[12] Axelson M., Björkhem I., Reihnér E., Einarsson K. (1991). The plasma level of 7 alpha-hydroxy-4-cholesten-3-one reflects the activity of hepatic cholesterol 7 alpha-hydroxylase in man. FEBS Lett.

[13] Schneider K.M., Candels L.S., Hov J.R., Myllys M., Hassan R., Schneider C.V. (2021). Gut microbiota depletion exacerbates cholestatic liver injury via loss of FXR signalling. Nat Metab.

[14] D’Amato D., De Vincentis A., Malinverno F., Viganò M., Alvaro D., Pompili M. (2021). Real-world experience with obeticholic acid in patients with primary biliary cholangitis. JHEP Rep.

[15] Kim W.R., Therneau T.M., Wiesner R.H., Poterucha J.J., Benson J.T., Malinchoc M. (2000). A revised natural history model for primary sclerosing cholangitis. Mayo Clin Proc.

[16] Scholz T., Karlsen T.H., Sanengen T., Schrumpf E., Line P.D., Boberg K.M. (2009). [Liver transplantation in Norway through 25 years]. Tidsskr Nor Laegeforen.

[17] Andersen I.M., Fosby B., Boberg K.M., Clausen O.P.F., Jebsen P., Melum E. (2015). Indications and outcomes in liver transplantation in patients with primary sclerosing cholangitis in Norway. Transpl Direct.

[18] Harrell F.E. (2021).

[19] Galman C., Angelin B., Rudling M. (2011). Pronounced variation in bile acid synthesis in humans is related to gender, hypertriglyceridaemia and circulating levels of fibroblast growth factor 19. J Intern Med.

[20] Mueller M., Thorell A., Claudel T., Jha P., Koefeler H., Lackner C. (2015). Ursodeoxycholic acid exerts farnesoid X receptor-antagonistic effects on bile acid and lipid metabolism in morbid obesity. J Hepatol.

[21] Zweers S.J., de Vries E.M., Lenicek M., Tolenaars D., de Waart D.R., Koelfat K.V.K. (2017). Prolonged fibroblast growth factor 19 response in patients with primary sclerosing cholangitis after an oral chenodeoxycholic acid challenge. Hepatol Int.

[22] Li Z., Lin B., Lin G., Wu Y., Jie Y., Li X. (2017). Circulating FGF19 closely correlates with bile acid synthesis and cholestasis in patients with primary biliary cirrhosis. PLoS One.

[23] Li Z., Liu Y., Yang F., Pang J., Wu Y., Chong Y. (2020). Dysregulation of circulating FGF19 and bile acids in primary biliary cholangitis-autoimmune hepatitis overlap syndrome. Biomed Res Int.

[24] Trauner M., Gulamhusein A., Hameed B., Caldwell S., Shiffman M.L., Landis C. (2019). The nonsteroidal farnesoid X receptor agonist cilofexor (GS-9674) improves markers of cholestasis and liver injury in patients with primary sclerosing cholangitis. Hepatology.

[25] Hirschfield G.M., Chazouillères O., Drenth J.P., Thorburn D., Harrison S.A., Landis C.S. (2019). Effect of NGM282, an FGF19 analogue, in primary sclerosing cholangitis: a multicenter, randomized, double-blind, placebo-controlled phase II trial. J Hepatol.

[26] Jansen P.L., Ghallab A., Vartak N., Reif R., Schaap F.G., Hampe J. (2017). The ascending pathophysiology of cholestatic liver disease. Hepatology.

[27] Al-Khaifi A., Rudling M., Angelin B. (2018). An FXR agonist reduces bile acid synthesis independently of increases in FGF19 in healthy volunteers. Gastroenterology.

