# Supplementary material for: Suppression of bile acid synthesis as a tipping point in the disease course of primary sclerosing cholangitis
Source: JHEP Rep. 2022 Aug 18;4(11):100561. doi: 10.1016/j.jhepr.2022.100561 (PMC9513776; doi:10.1016/j.jhepr.2022.100561)
Supplement: Multimedia component 1 [file mmc1.pdf]

Suppression of bile acid synthesis as a tipping point in the disease course of primary sclerosing cholangitis

Peder Rustøen Braadland, Kai Markus Schneider, Annika Bergquist, Antonio Molinaro, Anita Lövgren-Sandblom, Marcus Henricsson, Tom Hemming Karlsen, Mette Vesterhus, Christian Trautwein, Johannes Roksund Hov, Hanns-Ulrich Marschall

Table of contents

Supplementary materials and methods..2

Table S1..3

Table S2..4

Table S3..5

Fig. S1..6

Fig. S2..7

Supplementary materials and methods

UPLC-MS/MS

Concentrations of individual bile acids and C4 were determined using ultra-performance liquid chromatography-tandem mass spectrometry (UPLC-MS/MS) platforms. The Norwegian plasma samples were analysed using a QTRAP 5500 instrument (Sciex, Toronto, Canada) at the Wallenberg Laboratory (Sahlgrenska University Hospital, Gothenburg, Sweden) according to (i), and the Swedish samples were analysed using a Waters Xevo-TQ-XS instrument (Waters, Milford, UK) at the Department of Clinical Chemistry at Karolinska University Hospital (Huddinge, Stockholm, Sweden) according to (ii).

Previous comparisons of normal and cholestatic samples in both labs have not shown any significant differences in bile acids and C4 in matched human serum and plasma samples.

(i): Norwegian samples. Bile acids and C4 were extracted from 50 µL plasma using protein precipitation with 10 volumes of internal bile acid and C4 standard-containing methanol. After vortexing and centrifugation, the supernatant was evaporated and reconstituted in 200 µL methanol:water (1:1). Bile acids (5 µL injected) were separated using water with 7.5 mM ammonium acetate and 0.019% formic acid at a pH 4.5 as mobile phase A, and acetonitrile with 0.1% formic acid as phase B. The separation was made using gradient elution on a Kinetex C18 column (2.1x100 mm with 1.7 µm particles) (Phenomenex, Torrance, CA, USA) kept at 60 ºC. The gradient started with 1 minute of isocratic elution with 25% B, which was gradually increased to 35% over the next 4 minutes. During the next 9.5 minutes, B was increased from 35% to 95%. After one minute of isocratic elution, the gradient was quickly returned to 25% B and the column was equilibrated for 2.5 min to give a total runtime of 18 minutes per sample. The flow rate was 400 µL/min. Detection was made on a QTRAP 5500 instrument (Sciex, Toronto, Canada) with multiple reaction monitoring (MRM) in negative (anion) mode for bile acids and in a subsequent positive (cation) mode run for C4.

(ii): Swedish samples. Bile acids were extracted from 50 µL serum using protein precipitation with 25 ng internal bile acid standard mix in 40 µL methanol, vortexing, and 800 µL acetonitrile. After vortexing and centrifugation, the supernatant was evaporated and reconstituted in 240 µL methanol:water (1:1) with stepwise addition and vortexing of the solvents. Bile acids (5 µL injected) were separated using water with 5 mM ammonium acetate and 0.01% formic acid as mobile phase A, and methanol with 5 mM ammonium acetate and 0.01% formic acid as phase B. The separation was made using gradient elution on a Waters Acquity UPLC BEH C18 column (2.1x50 mm with 1.7 µm particles) (Waters AB, Solna, Sweden) kept at 45 ºC. The gradient started with 1 minute of isocratic elution with 19% B, which was increased to 35% over the next 4 minutes. During the next 13.5 minutes, B was increased from 35% to 100%. After one minute of isocratic elution, the gradient was quickly returned to 20% B and the column was equilibrated for 1.5 min to give a total runtime of 20 minutes per sample. The flow rate was 400 µL/min. Detection was made on a Waters Xevo-TQ-XS platform with MRM in negative (anion) mode for bile acids and in a positive (cation) mode run for C4 prepared as follows:

C4 was extracted from another 75 µL of serum using HybridSPE-96-well plates (Sigma-Aldrich AB, Stockholm, Sweden) for protein and phospholipid removal after adding 2.5 ng internal C4 standard in 200 µl acetonitrile with 1% formic acid and 9% 2-propanol, vortexing, addition of 100 µL acetonitrile. From vacuum-supported flow-through of the wells, 10 µL were injected on the same Waters Acquity UPLC BEH C18 column as above. Mobile phase A was 0.1% formic acid in water, mobile phase B was 0.1% formic acid in acetonitrile. The gradient was started with 50% B, direct after injection increased to 100% B, isocratic for 4 minutes and then returned 50% B. The total run time at 0.6 ml/min was 6 minutes.

Unlabeled and deuterium labelled internal standards were obtained from Sigma-Aldrich (Sweden, TCA, TUDCA, TCDCA, TDCA, TLCA, GCA, GCDCA, GDCA, GLCA, CA, UDCA, CDCA, DCA, LCA, C4), CDN isotopes (Quebec, Canada, d4-GCDCA, d4-GLCA, d4-GCA, d4-GUDCA, d4-CDCA, d4-UDCA, d4-LCA) and Toronto Research Chemicals (Downsview, Ontario, Canada, d4-TCA, d6-C4).

For the statistical analyses, bile acids were excluded from all analyses if they only were found in trace amounts or were absent in >96% of the samples in both sets. These included mostly hyodeoxycholic acid and conjugated and unconjugated muricholic acids.

Supplementary figures and tables

Table S1. Blood concentrations and interquartile ranges of C4, individual bile acids (imputed data) and their sums and ratios in healthy controls and PSC patients from the discovery and validation cohorts.

	**Healthy controls, N = 100**[1]	**Discovery, PSC, N = 191**[1]	**Validation, PSC, N = 139**[1]	**p**[2]
**Individual bile acid species, nmol/L**				
7α-hydroxy-cholesten-3-one (C4)	33 [17–52]	9 [3–27]	33 [13–60]	<0.001
Cholic acid (CA)	46 [20–266]	45 [20–107]	49 [13–151]	0.2
Chenodeoxycholic acid (CDCA)	94 [38–222]	49 [18–156]	119 [47–329]	0.001
Deoxycholic acid (DCA)	184 [84–321]	24 [5–72]	137 [43–372]	<0.001
Glyocholic acid (GCA)	203 [86–327]	3,037 [686–13,001]	785 [240–2,528]	<0.001
Glycochenodeoxycholic acid (GCDCA)	613 [321–1,091]	2,576 [1,135–9,386]	2,319 [686–6,545]	<0.001
Glycodeoxycholic acid (GDCA)	281 [142–602]	373 [163–1,061]	523 [166–1,167]	0.034
Glycolithocholic acid (GLCA)	15 [8–40]	16 [4–42]	54 [18–164]	0.4
Glycoursodeoxycholic acid (GUDCA)	70 [37–124]	283 [48–10,132]	1,575 [193–15,486]	<0.001
Hyocholic acid (HCA)	10 [6–14]	14 [8–29]	8 [5–16]	<0.001
Iso-ursodeoxycholic acid (isoUDCA)	61 [29–142]	34 [6–146]	175 [27–622]	0.003
Lithocholic acid (LCA)	6 [4–9]	6 [3–14]	64 [26–107]	0.9
Taurocholic acid (TCA)	37 [19–76]	2,025 [217–8,195]	243 [55–1,822]	<0.001
Taurochenodeoxycholic acid (TCDCA)	66 [36–113]	1,018 [168–4,694]	484 [116–1,820]	<0.001
Taurodeoxycholic acid (TDCA)	39 [18–89]	127 [35–339]	163 [32–312]	<0.001
Taurolithocholic acid (TLCA)	3 [2–8]	12 [3–27]	12 [6–23]	<0.001
Tauroursodeoxycholic acid (TUDCA)	2 [1–3]	47 [6–628]	185 [13–1,184]	<0.001
Ursodeoxycholic acid (UDCA)	21 [9–47]	26 [4–629]	835 [34–4,173]	0.13
**Sums and ratios**				
Primary bile acids, nmol/L	1,305 [680–2,139]	10,565 [2,639–36,344]	5,162 [2,056–17,967]	<0.001
Secondary bile acids, nmol/L	886 [494–1,312]	2,224 [545–14,870]	8,596 [1,937–27,719]	<0.001
Secondary/primary (ratio)	0.68 [0.43–1.02]	0.35 [0.07–1.08]	1.40 [0.39–3.45]	<0.001
Conjugated bile acids, nmol/L	1,534 [780–2,424]	14,645 [4,097–53,872]	10,467 [3,688–45,255]	<0.001
Deconjugated bile acids, nmol/L	569 [316–1,077]	440 [166–1,788]	2,234 [713–6,743]	0.4
Deconjugated/conjugated (ratio)	0.36 [0.20–0.80]	0.05 [0.01–0.16]	0.20 [0.05–0.63]	<0.001
Total bile acids, nmol/L	2,230 [1,406–3,422]	17,772 [4,762–55,606]	14,935 [6,099–52,085]	<0.001

[1]Median [25%–75%],
[2]Wilcoxon rank-sum test, discovery cohort vs healthy controls

Table S2. Blood concentrations and interquartile ranges of C4, individual bile acids and their sums and ratios in patients with PSC either naïve to or treated with UDCA in the discovery and validation cohorts.

	**Discovery cohort, N=191**			**Validation cohort, N=139**		
	**UDCA-naïve, N = 120**[1]	**UDCA-treated, N = 71**[1]	**p**[2]	**UDCA-naïve, N = 32**[1]	**UDCA-treated, N = 107**[1]	**p**[2]
**Individual bile acid species**						
C4	9 [3–32]	8 [2–22]	0.3	34 [22–67]	31 [8–58]	0.2
CA	45 [24–126]	44 [19–95]	0.3	57 [12–212]	45 [16–145]	0.6
CDCA	42 [17–127]	60 [19–231]	0.14	85 [48–199]	132 [47–358]	0.2
DCA	26 [5–100]	23 [6–56]	0.5	123 [40–268]	147 [43–382]	0.4
GCA	1,906 [520–8,898]	6,928 [1,391–14,230]	0.007	636 [203–1,705]	940 [271–2,870]	0.2
GCDCA	2,058 [927–9,314]	5,399 [1,884–9,942]	0.009	1,684 [778–2,859]	3,159 [686–10,601]	0.051
GDCA	328 [146–885]	516 [265–1,185]	0.025	267 [126–512]	679 [214–1,488]	<0.001
GLCA	11 [4–27]	34 [6–116]	<0.001	36 [12–62]	73 [19–260]	0.003
GUDCA	73 [29–210]	17,171 [6,783–24,675]	<0.001	58 [16–164]	5,412 [849–19,519]	<0.001
HCA	14 [7–28]	15 [8–29]	0.5	5 [5–11]	10 [6–16]	0.003
IsoUDCA	14 [1–51]	127 [50–348]	<0.001	15 [3–34]	259 [104–933]	<0.001
LCA	4 [3–7]	13 [7–26]	<0.001	30 [13–42]	75 [35–124]	<0.001
TCA	1,461 [183–8,518]	2,468 [367–7,794]	0.7	173 [67–1,377]	256 [52–1,989]	0.8
TCDCA	774 [153–5,906]	1,604 [470–3,904]	0.5	367 [156–1,223]	486 [98–1,978]	0.7
TDCA	133 [34–346]	114 [36–279]	0.7	150 [51–262]	164 [27–410]	0.8
TLCA	8 [3–19]	16 [6–49]	0.002	9 [3–15]	13 [7–26]	0.023
TUDCA	10 [2–43]	1,576 [323–5,042]	<0.001	3 [1–16]	498 [50–1,463]	<0.001
UDCA	6 [1–21]	1,290 [311–2,875]	<0.001	7 [3–19]	1,747 [503–5,557]	<0.001
**Sums and ratios**						
Primary	7,146 [2,333–37,260]	19,030 [4,782–35,421]	0.11	3,889 [2,037–10,051]	6,299 [2,073–22,382]	0.2
Secondary	821 [401–1,830]	22,116 [11,369–35,462]	<0.001	1,023 [655–1,437]	13,854 [5,158–35,520]	<0.001
Secondary/ primary	0.14 [0.04–0.30]	1.22 [0.88–2.27]	<0.001	0.3 [0.1–0.5]	2.0 [0.9–4.9]	<0.001
Conjugated	7,924 [2,445–37,553]	38,639 [12,328–65,098]	<0.001	3,892 [2,448–10,514]	17,258 [5,228–63,985]	<0.001
Deconjugated	199 [99–509]	2,309 [696–5,011]	<0.001	535 [248–962]	3,989 [1,435–7,969]	<0.001
Deconjugated /conjugated	0.03 [0.01–0.13]	0.08 [0.02–0.17]	0.011	0.14 [0.03–0.30]	0.22 [0.06–0.73]	0.12
Total bile acids	8,959 [2,839–38,364]	41,898 [14,947–71,898]	<0.001	4,639 [3,296–10,779]	20,526 [9,794–68,610]	<0.001

[1]Median [25%–75%]
[2]Wilcoxon rank-sum test

Table S3. Baseline characteristics of patients included in the survival (time-to-event) analyses.

Characteristic	Discovery, Norway N = 167	Validation, Sweden N = 135
**Sex, female**	36 (22%)	44 (32%)
**Age at sampling**	39 [16–71]	42 [21–77]
**Inflammatory bowel disease, any**	126 (76%)	100 (74%)
Ulcerative colitis	83 (67%)	82 (82%)
Crohn's disease	26 (21%)	16 (16%)
Indeterminate colitis	15 (12%)	1 (1%)
Unknown	0 (0%)	1 (1%)
**Ursodeoxycholic acid treatment**	64 (38%)	99 (77%)
Missing	0	7 (5%)
**Hepatobiliary cancer, any**	0 (0%)	0 (0%)
**Variceal bleeding**	3 (1.8%)	2 (1.4%)
**Ascites**	16 (10%)	0 (0%)
**Encephalopathy**	1 (0.6%)	0 (0%)
**Mayo PSC score [IQR]**	0.09 [-0.50–0.92]	0.04 [-0.58–0.78]
**AOM PSC score [IQR]**	1.70 [1.24–2.32]	1.78 [1.44–2.31]

Continuous variables are shown as median [interquartile range]. Abbreviations: AOM=Amsterdam-Oxford model, CI=confidence interval.

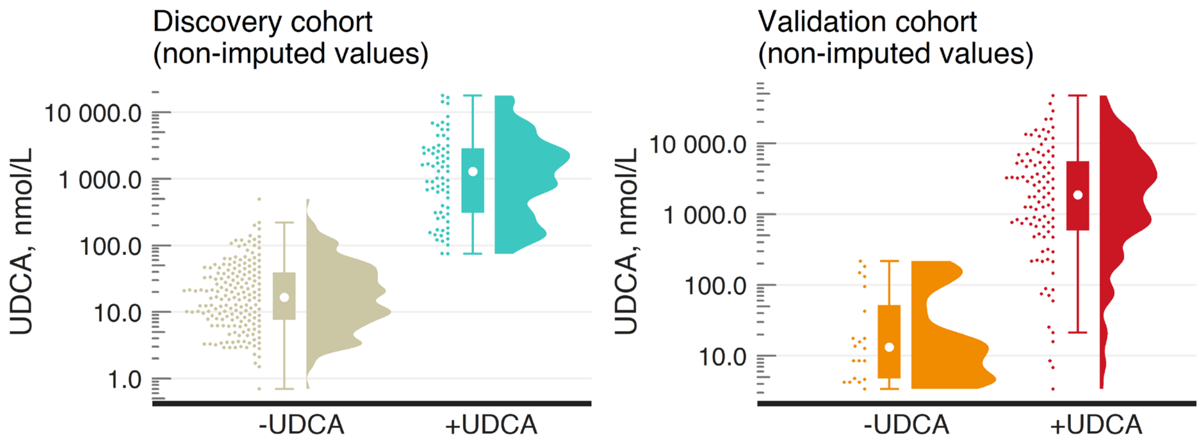

Fig. S1. Correspondence between circulating UDCA level and UDCA use as indicated in patient journals. Blood UDCA concentration (imputed values are not shown) by UDCA treatment as indicated in journal records for patients in the discovery (left panel) and validation (right panel) cohorts.

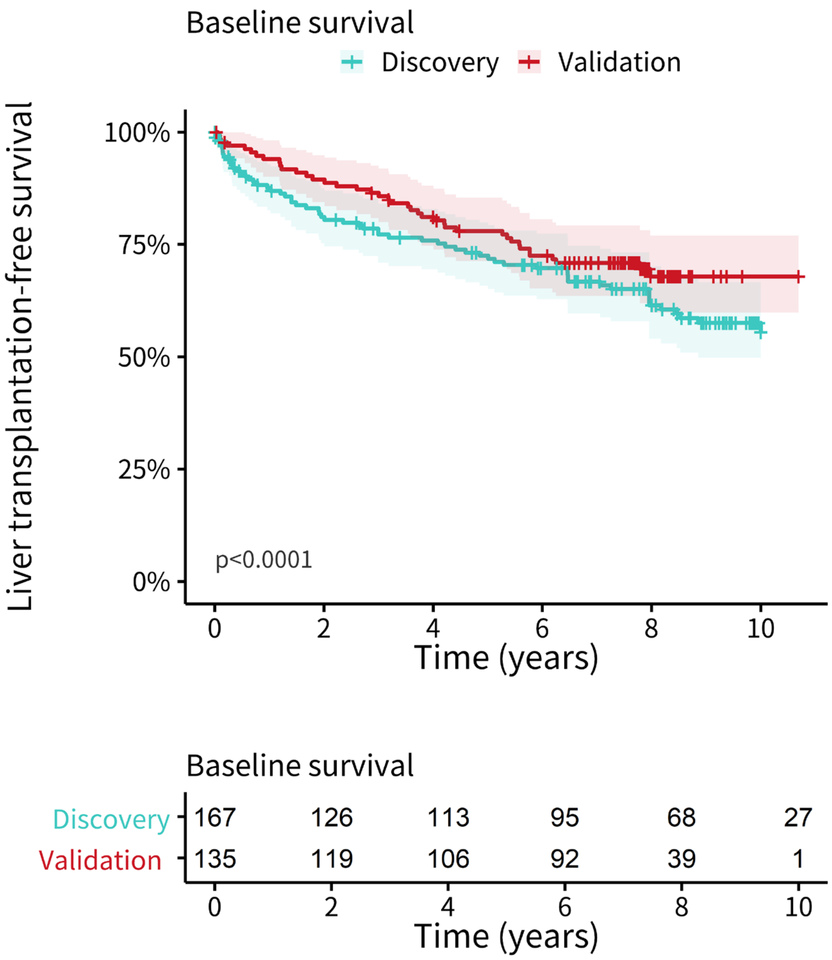

Fig. S2. Baseline liver-transplantation free survival with 95% confidence intervals, calculated using the Kaplan-Meier method, among eligible patients in the discovery and validation cohorts. The number of patients at risk at each indicated time point is shown below the plot.
