# Supplementary material for: Suppression of bile acid synthesis as a tipping point in the disease course of primary sclerosing cholangitis
Source: JHEP Rep. 2022 Aug 18;4(11):100561. doi: 10.1016/j.jhepr.2022.100561 (PMC9513776; doi:10.1016/j.jhepr.2022.100561)
Supplement: Multimedia component 2 [file mmc2.pdf]

JHEP Reports

CTAT methods

Tables for a "Complete, Transparent, Accurate and Timely account" (CTAT) are now mandatory for all revised submissions. The aim is to enhance the reproducibility of methods.

- Only include the parts relevant to your study
- Refer to the CTAT in the main text as 'Supplementary CTAT Table'
- Do not add subheadings
- Add as many rows as needed to include all information
- Only include one item per row

If the CTAT form is not relevant to your study, please outline the reasons why:

1.1 Antibodies

Name	Citation	Supplier	Cat no.	Clone no.

1.2 Cell lines

Name	Citation	Supplier	Cat no.	Passage no.	Authentication test method

1.3 Organisms

Name	Citation	Supplier	Strain	Sex	Age	Overall n number

1.4 Sequence based reagents

Name	Sequence	Supplier

1.5 Biological samples

Description	Source	Identifier
Norwegian discovery cohort (patients with PSC, N=191, and healthy donors, N=100)	Plasma. Oslo University Hospital, Rikshospitalet (Oslo, NO) 2008-2015. Norwegian Bone Marrow Donor Registry	NoPSC biobank, Oslo, Norway 2011/2572, 2015/2140
Swedish validation cohort (only patients with PSC, N=139)	Serum. Karolinska University Hospital, (Stockholm, SE), 2008-2012	2018/1111-32

1.6 Deposited data

Name of repository	Identifier	Link

1.7 Software

Software name	Manufacturer	Version
R	R core team	v. 4.1.1

1.8 Other (*e.g.* drugs, proteins, vectors etc.)

Unlabeled and deuterium-labelled internal standards (UPLC-MS/MS)	Sigma-Aldrich	
CDN standards	Quebec, Canada<br>Toronto Research Chemicals, Canada	

1.9 Please provide the details of the corresponding methods author for the manuscript:

Hanns-Ulrich Marschall,
Department of Molecular and Clinical Medicine / Wallenberg Laboratory
Sahlgrenska Academy and University Hospital
S-413 45 Gothenburg, Sweden
hanns-ulrich.marschall@gu.se

2.0 Please confirm for randomised controlled trials all versions of the clinical protocol are included in the submission. These will be published online as supplementary information.
